# Supplementary material for: Health Potential of Clery Strawberries: Enzymatic Inhibition and Anti-Candida Activity Evaluation
Source: Molecules. 2021 Mar 19;26(6):1731. doi: 10.3390/molecules26061731 (PMC8003815; doi:10.3390/molecules26061731)
Supplement: Supplementary file 1 [file molecules-26-01731-s001.zip › molecules-1131566-supplementary.pdf]

Health potential of Clery strawberries: enzymatic inhibition and anti-*Candida* activity evaluation

Francesco Cairone[a,†], Giovanna Simonetti[b,†] Anastasia Orekhova[c], Maria Antonietta Casadei[a], Gokhan Zengin[d], Stefania Cesa[a]

[a]*Dipartimento di Chimica e Tecnologie del Farmaco, Università degli Studi di Roma "La Sapienza", Piazzale Aldo Moro 5, 00185 Roma, Italy*

[b]*Dipartimento di Biologia Ambientale, Università degli Studi di Roma "La Sapienza" P.le Aldo Moro5, 00185 Rome, Italy*

[c]*Dipartimento di Sanità Pubblica e Malattie infettive, Università degli Studi di Roma "La Sapienza" P.le Aldo Moro 5, 00185 Rome, Italy*

[d]*Department of Biology, Science Faculty, Selcuk University, 42130 Konya, Turkey*

† The authors contributed equally

*Corresponding author: Stefania Cesa

Address: Department of Drug Chemistry and Technology, Università degli Studi di Roma "LaSapienza", Piazzale Aldo Moro 5, 00185 Rome, Italy

Tel.: + 39 0649913198; fax: + 39 0649913133

E-mail address: stefania.cesa@uniroma1.it

Table S1. Colorimetric CIEL*a*b* parameters of Clery graft on *Fragaria vesca* strawberryhomogenates. Reported results represent the mean value ± SD from four experiments.

Sample	L*	a*	b*	C^*_{ab}	h_{ab}
**M graft**	36.0 ± 0.3	18.5 ±1.2	8.8 ± 0.7	20.5 ± 1.4	25.5 ± 0.2
**U graft**	36.5 ± 0.3	17.9 ± 0.3	8.1 ± 0.2	19.6 ± 0.3	24.4 ± 0.3
**MP graft**	37.6 ± 0.4	18.5 ± 0.8	8.3 ± 0.3	20.3 ± 0.9	24.0 ± 0.4
**UP graft**	37.6 ± 0.6	17.6 ± 0.6	7.3 ± 0.5	19.1 ± 0.7	22.6 ± 0.9
**BM graft**	44.0 ± 0.2	22.4 ± 0.6	9.9 ± 0.9	24.5 ± 0.9	23.8 ± 1.7
**BU graft**	43.0 ± 0.7	21.9 ± 0.4	11.0 ± 0.3	24.5 ± 0.5	26.5 ± 0.3
**BMP graft**	41.5 ± 0.3	18.2 ± 1.2	7.5 ± 0.8	19.6 ± 1.4	22.3 ± 0.9
**BUP graft**	41.1 ± 0.9	18.0 ± 0.4	8.0 ± 0.2	19.7 ± 0.4	24.1 ± 0.1
**WM graft**	35.9 ± 0.3	18.8 ± 0.4	8.7 ± 0.8	20.8 ± 0.8	24.7 ± 1.6
**WU graft**	38.5 ± 0.2	21.5 ± 1.2	9.5 ± 1.9	23.6 ± 1.9	24.0 ± 3.2
**PM graft**	37.2 ± 2.2	20.0 ± 0.1	9.4 ± 0.3	22.1 ± 0.2	25.0 ± 0.7
**PU graft**	39.2 ± 3.1	21.7 ± 0.8	9.5 ± 0.6	23.7 ± 0.9	23.5 ± 0.8

Table S2. HPLC-DAD quantitative analysis of Clery graft samples.

	**M graft**	**U graft**	**MP graft**	**UP graft**	**BM graft**	**BU graft**	**BMP graft**	**BUP graft**	**WM graft**	**WU graft**	**PM graft**	**PU graft**
**Catechin**	59.7	113.2	50.3	58.3	125.7	223.7	64.1	52.8	68.1	63.4	76.4	174.9
**Epicatechin**	277.9	300.8	402.2	268.6	365.2	374.4	257.8	197.4	154.2	285.3	197.7	217.5
***p*-Coumaric acid**	12.9	13.2	13.6	13.6	nd	nd	4.5	8.9	7.2	18.7	8.6	5.7
**Ferulic acid and derivatives***	209.3	242.5	222.2	166.9	145.7	219.8	109.1	82.7	85.5	97.9	167.4	170.3
**Flavonols****	8.6	4.2	5.4	3.9	3.1	7.6	2.1	2.9	2.4	9.3	4.8	18.1
**Anthocyanins*****	91.8	78.4	70.9	65.9	128.1	99.1	41.8	63.1	37.5	77.8	183.6	190.1

Results are expressed in µg/g of fresh weight. The RSD value, evaluated on triplicates, was < 5%. *Expressed as ferulic acid. ** Expressed as quercetin-3-D-galactoside. *** Expressed as pelargonidin–3–glucoside. nd = not detected
